# Supplementary material for: Coordinated expansion of CD163+ monocytes and immature CD177+ neutrophils marks severe neurotoxicity after CD19 CAR T cell therapy
Source: bioRxiv. 2026 Jul 9:2026.07.07.737099. Preprint. [Version 1] doi: 10.64898/2026.07.07.737099 (PMC13371044; doi:10.64898/2026.07.07.737099)
Supplement: Supplement 2 [file media-2.pdf]

Supplemental Table 1

**Characteristic**	Discovery Cohort<br>N = 33[1]	Validation Cohort<br>N = 12[1]
**Age at CAR T-cell infusion (years)**		
Median (Q1, Q3)	67.1 (57.6, 71.5)	71.7 (68.0, 76.8)
Min - Max	33.5 - 84.3	64.5 - 81.9
**Sex**		
Female	14 (42%)	6 (50%)
Male	19 (58%)	6 (50%)
**Diagnosis**		
Acute lymphoblastic leukemia	1 (3%)	0 (0%)
Follicular lymphoma	1 (3%)	2 (17%)
Large B-cell lymphoma	29 (88%)	5 (42%)
Mantle cell lymphoma	2 (6%)	5 (42%)
**CAR T-cell product**		
Axicabtagene ciloleucel	18 (55%)	0 (0%)
Brexucabtagene autoleucel	3 (9%)	0 (0%)
Lisocabtagene maraleucel	6 (18%)	12 (100%)
Tisagenlecleucel	6 (18%)	0 (0%)

[1] n (%)

Supplemental Table 2

Target	Clone	Metal Isotope	Source	Part number
CD45	HI3O	89 Y	Standard Biotools	3089003
CD4	RPA-T4	110 Cd	BioLegend	300502
CD8a	RPA-T8	111 Cd	BioLegend	301002
CD7	CD7-6B7	112 Cd	BioLegend	343102
CD63	H5C6	113 Cd	BD Pharmingen	556019
BDCA3/CD141	1A4	114 Cd	BD Pharmingen	230282
CD66b	G10F5	116 Cd	BioLegend	305102
CD10	HI10a	141 Pr	BioLegend	312202
CD101	BB27	142 Nd	ThermoFisher	14-1019-82
CD123	6H6	143 Nd	Standard Biotools	3143014B
TCRgdAnti-FITC	5A6.E9/FIT-22	144 Nd	ThermoFischer/Standard Biotools	MHGD01/3144006B
CD163	GHI/61	145 Nd	Standard Biotools	3145010B
CD69	FN50	146 Nd	BioLegend	310902
CD16	3G8	148 Nd	Standard Biotools	3148004B
CD24	ML5	149 Sm	BioLegend	311102
FcεR1α	AER-37	150 Sm	Standard Biotools	3150027B
CD226	DNAM-1	151 Eu	BioLegend	338302
CD62L	DREG-56	152 Sm	BioLegend	304802
CD38	HIT2	154 Sm	BioLegend	303502
CD95	DX2	155 Gd	BioLegend	305602
TCRva7.2	3C10	156 Gd	BioLegend	351702
CD15	W6D3	157 Gd	BioLegend	323002
CD235AB	HIR2	160 Dy	BioLegend	306602
CD161	HP-3G10	161 Dy	BioLegend	339902
CD71	OKT-9	162 Dy	ThermoFisher	14-0719-82
CD1c	L161	163 Dy	BioLegend	331502
CD64	10.1	165 Ho	BioLegend	305002
CD19	HIB19	168 Er	BioLegend	302202
CD45RA	HI100	169 Tm	Standard Biotools	3169008B
PDL1/CD274	29E.2A3	170 Er	BioLegend	329702
CD56	HCD56	171 Yb	BioLegend	318345
CD193	5E8	172 Yb	BioLegend	310702
HLA-DR	L243	173 Yb	Standard Biotools	3173005B
CD89	A59	174 Yb	Standard Biotools	3174012B
CD3	UCHT1	194 Pt	BioLegend	300402
CD5	UCHT2	195 Pt	BioLegend	300602
CD14	HCD14	198 Pt	BioLegend	325602
CD11b	ICRF44	209 Bi	Standard Biotools	3209003B
CD11c	3.9	106 Cd	BioLegend	301602
pSTAT1	58D6	153 Eu	Standard Biotools	3153003A
pSTAT3	4/P-STAT3	158 Gd	Standard Biotools	3158005A
pSTAT5	47	147 Sm	Standard Biotools	3147012A
pCREB	87G3	176 Lu	Standard Biotools	3176005A
prpS6	N7-548	175 Lu	Standard Biotools	3175009A
pMAPKAPK2	27B7	159 Tb	Standard Biotools	3159010A
pNFκB	K10x	166 Er	Standard Biotools	3166006A
pERK1/2	D1314.4E	167 Er	Standard Biotools	3167005A
Ikb	L35A5	164 Dy	Standard Biotools	3164004A

Supplemental Table 3

Assay	Vendor	CATALOG #	Format	Analyte	Sample dilution
A	MSD	K15067M-2	UPLEX Custom Biomarker	GM-CSF, IL-10, MIP1a, IL-2, IL-22, IL-12p70, IL-5, MCP4, IL-4, IL-13	1
B	MSD	K15067M-2	UPLEX Custom Biomarker	IFNy, IL-18, IL-1RA, IL-15, IL-6, IL-8, IP-10, MCP1, sIL-2Ra, MIG	4
C	MSD	K151198D-2	V-PLEX Vascular Injury	CRP, SAA, ICAM-1, VCAM-1	10,000
D	MSD	K151P3S-2	SPLEX	IFN-a2a	1
E	MSD	K151ADQS-2	SPLEX	IL-3	1
F	MSD	K151ADRS-2	SPLEX	IFNb	2
G	R&D	DY119	ELISA	IL-18BP	40 or 200
H	MSD	K151AEM-2	UPLEX Custom Immuno-oncology	MMP9(total), S100A12	100
I	MSD	K1514ER-2	RPLEX	MPO	20
